# Resynthesis of Damaged Fe-S Cluster Proteins Protects *Aspergillus fumigatus* Against Oxidative Stress in the Absence of Mn-Superoxide Dismutase

**DOI:** 10.3390/jof10120823

**Published:** 2024-11-27

**Authors:** Klaudia Pákozdi, Károly Antal, Kitti Pázmándi, Márton Miskei, Zsuzsa Szabó, István Pócsi, Tamás Emri

**Affiliations:** 1 Department of Molecular Biotechnology and Microbiology, Institute of Biotechnology, Faculty of Science and Technology, University of Debrecen, H-4032 Debrecen, Hungary; pakozdi.klaudia@science.unideb.hu (K.P.); szabo_zsuzsi09@yahoo.com (Z.S.); 2 Doctoral School of Nutrition and Food Sciences, University of Debrecen, H-4032 Debrecen, Hungary; 3 Department of Zoology, Eszterházy Károly Catholic University, H-3300 Eger, Hungary; antalk2@gmail.com; 4 Department of Immunology, Faculty of Medicine, University of Debrecen, H-4032 Debrecen, Hungary; pazmandi.kitti@med.unideb.hu; 5 HUN-REN–UD Fungal Stress Biology Research Group, H-4032 Debrecen, Hungary; miskeim@gmail.com

**Keywords:** *Aspergillus fumigatus*, iron homeostasis, mitochondrion, Mn-superoxide dismutase, oxidative stress, RNA sequencing

## Abstract

The importance of manganese superoxide dismutase (Mn-SOD), an evolutionarily ancient metalloenzyme that maintains the integrity and function of mitochondria, was studied in oxidative stress-treated *Aspergillus fumigatus* cultures. Deletion of the Mn-SOD gene (*sodB*) increased both the menadione sodium bisulfite (MSB)-elicited oxidative stress and the deferiprone (DFP)-induced iron limitation stress sensitivity of the strain. Moreover, DFP treatment enhanced the MSB sensitivity of both the gene deletion mutant and the reference strain. The lack of SodB also increased the susceptibility of conidia to killing by human macrophages. Concurring with the stress sensitivity data, RNS sequencing data also demonstrated that the deletion of *sodB* largely altered the MSB-induced oxidative stress response. The difference between the oxidative stress responses of the two strains manifested mainly in the intensity of the response. Importantly, upregulation of “Ribosome protein”, “Iron uptake”, and “Fe-S cluster assembly” genes, alterations in the transcription of “Fe-S cluster protein” genes, and downregulation of “Heme binding protein” genes under MSB stress were characteristic only for the Δ*sodB* gene deletion mutant. We assume that the elevated superoxide level generated by MSB treatment may have destroyed Fe-S cluster proteins of mitochondria in the absence of SodB. This intensified the resynthesis of Fe-S cluster proteins, which was accompanied with enhanced translation and iron acquisition, leading to increased DFP sensitivity.

## 1. Introduction

The evolutionary ancient metalloenzymes manganese superoxide dismutases (MnSODs) maintain yeast and filamentous fungi mitochondrial integrity and function [1,2,3,4,5]. In *Aspergillus nidulans*, deletion of the *mnSOD/sodB* gene increased sensitivity to menadione (a redox-active 1,4-naphthoquinone that increases intracellular superoxide levels) and PAF (*Penicillium chrysogenum* antifungal protein, known to cause apoptotic cell death in susceptible fungi), reduced respiration rates in carbon-starved cultures, affected growth rate, antioxidant enzyme activities, and sterigmatocystin production depending on glucose availability in the culture medium, and also affected conidial yield, viability, and heat tolerance [3]. In the aflatoxin producer *Aspergillus flavus*, deletion of the orthologous MnSOD gene resulted in slow growth phenotypes under various culture conditions and negatively affected aflatoxin B1 production [6]. According to Lambou et al. [7], *Afsod2* was expressed intensely in conidia and (at later time points) in growing mycelia of the human pathogenic fungus *Aspergillus fumigatus*. *Afsod2* was upregulated by menadione and heat treatments and, not surprisingly, the Δ*Afsod2* mutant was characterized by menadione- and heat-sensitive phenotypes [7]. AfSod2 also plays a pivotal role in the resistance against reactive oxygen and reactive nitrogen species (ROS and RNS) generated by *Pseudomonas aeruginosa*-derived phenazines [8]. In summary, fungal MnSODs are important players in the maintenance of both conidia and vegetative tissues and in the defense against various types of environmental stress, and they 0can also modulate mycotoxin production in Aspergilli in a species-specific manner.

Global transcriptomic changes caused by the ablation of MnSOD genes showed different patterns in various fungal species with some overlaps. In the baker’s yeast *Saccharomyces cerevisiae*, *SOD2* deficiency downregulated several metabolic processes and oxidative stress response genes reflecting a global suppressive response to reduced mitochondrial activity [9]. In the filamentous fungus *Podospora anserina*, which is a widely used model organism to study the molecular biological background of senescence, autophagy (especially mitophagy) was upregulated together with mitochondrial biogenesis, mitochondrial metabolism, and mitochondrial respiration genes to compensate for the loss of the *Pasod3* MnSOD gene [10]. In *A. nidulans*, the differences between the transcriptomes of glucose-supplemented unstressed and menadione-exposed control and Δ*sodB* cultures were relatively small, and this model fungus seemed to compensate easily for the loss of *sodB* with antioxidative enzymes and intracellular antioxidants and the downregulation of mitochondrial functions [11]. Importantly, menadione exposures concomitantly upregulated mitochondrial assembly processes including iron–sulfur cluster assembly genes, as well as genes involved in the autophagy (mitophagy) and/or disassembly of mitochondrion in both strains [11].

Any perturbation of the iron homeostasis (either iron-limitation or iron-excess) of *A. fumigatus* may result in disturbances in mitochondrial and other cellular functions [12]. In addition, mitochondrial iron–sulfur cluster assembly is essential for iron-sensing, and its blockage leads to an iron starvation response [13]. It is noteworthy that genetic modulation of the reductive iron assimilation (RIA) and siderophore-mediated iron acquisition pathways or their regulation typically elevate intracellular ROS concentrations, giving rise to oxidative stress sensitive phenotypes [14,15,16,17,18]. Targeting of either the iron acquisition system or the antioxidant defense system with drugs or drug candidate molecules has provided us with encouraging results to initiate further research in this area [19,20,21].

Deferiprone (DFP) is an iron chelator used to treat iron overload in thalassemia major [22]. It is able to remove iron from transferrin and lactoferrin but can also enter mammalian cells (even mitochondria) and form Fe^3+^ complexes in them [23,24]. DFP, like most iron chelators, is not fully specific for Fe^3+^; it can also be used to reduce Cu^2+^ or Zn^2+^ toxicity [24]. DFP inhibited the metabolic activity of biofilm-forming *A. fumigatus* cultures or pre-formed biofilms, and this effect was a consequence of iron chelation [25,26]. Transcriptomic and physiological data showed that DFP treatment was able to induce an iron starvation stress response in *A. fumigatus* without affecting ROS production and copper homeostasis, but the treatment upregulated Zn^2+^ transporter genes [27].

In this study, we demonstrate that deletion of *A. fumigatus Afsod2* (*sodB*) resulted in a menadione sensitive phenotype in both surface agar- and liquid-submerged cultures. Furthermore, the *sodB* gene deletion increased the sensitivity of the fungus to iron depletion induced by DFP. Moreover, the combination of menadione-triggered superoxide stress with DFP treatment was deleterious for both the wild type and the Δ*sodB* strains.

## 2. Materials and Methods

### 2.1. Strains and Culturing Conditions

The *A. fumigatus akuB^ku80^* parental strain and the IP345 Δ*sodB* (*sod2*) mitochondrial manganese superoxide dismutase (Mn-SOD) mutant were studied [7]. For all experiments, conidia freshly collected from cultures and incubated at 37 °C for 6 days on Barratt’s minimal nitrate agar plates [28] were used.

For submerged cultivation, aliquots of modified Barratt’s minimal broth (100 mL in 500 mL Erlenmeyer flasks) were inoculated with 5 × 10^7^ conidia. Cultures were incubated for 20 h at 37 °C and at a 3.7 Hz shaking frequency (approx. 220 rpm). The modified Barratt’s minimal broth was made with 20 g/L glucose (instead of 10 g/L) and 0.92 g/L diammonium-tartrate (instead of 6 g/L NaNO_3_). The 20 h, exponentially growing cultures were treated with 6 mM menadione sodium bisulfite (MSB) or left untreated. For isolation of total RNA and measurement of superoxide formation and SOD activities, mycelium samples were taken 0.5 h after stress treatment.

For surface cultivation, modified Barratt’s minimal agar plates containing only 10 g/L glucose was used. In some experiments, media were supplemented with 0.5, 1, 15 μM MSB, and/or 0.8 mM deferiprone (DFP) as an iron chelator. In the case of “DFP containing” media, the trace element solution, used for preparing the modified Barratt’s minimal agar plates, did not contain any FeSO_4_. Some of the media were point-inoculated with 100,000 conidia (5 μL suspension [3]) and the colony diameters were recorded after incubation at 37 °C for 5 days. Some other media were inoculated by spreading out 10^6^ conidia (100 μL suspension) on their surface. In the center of each plate, a 6 mm diameter well was drilled, and the cultures were pre-incubated at 37 °C for 5 days. After pre-incubation, 50 μL of 12 mM MSB solution was pipetted into the wells and cultures were further incubated at 37 °C for 4 days; the diameters of the inhibition zones were then measured.

### 2.2. Determining Formation of Superoxide (Et-Test) and Measuring Specific SOD Activities

Intracellular formation of superoxide was quantified with the Et-test [29]. Aliquots (20 mL) from the cultures were incubated with 0.01 mM dihydroethidium (1 h, 37 °C, 220 rpm). Mycelia from 5 mL samples were filtrated, washed with ice-cold distilled water, and resuspended in ice-cold 5 *w*/*v*% 5′-sulfosalicylic acid. After incubation at 4 °C for 10 min, the samples were centrifuged (10,000 G, 10 min, 4 °C) and the ethidium (Et) formed was determined spectrofluorimetrically (λ_ext_ = 488 nm, λ_em_ = 610 nm) from the supernatant. The dry cell mass (DCM) of the cultures were determined from a further 5 mL sample, and the Et content was expressed as pmol Et/g DCM.

The spectrophotometric rate assay developed by Oberley and Spitz [30] was used to quantify total SOD activities in the samples. The reaction mixture contained 10 *v*/*v*% cell-free extract, 0.2 mM xantin, 0.01 Unit/L xantin oxidase (Merck Ltd., Budapest, Hungary), 1000 U/L catalase (Merck Ltd., Budapest, Hungary), 70 μM nitroblue tetrazolium (Merck Ltd., Budapest, Hungary), and 1.4 mM diethylenetriaminepentaacetic acid (DETAPAC) dissolved in 50 mM K-phosphate buffer (pH 7.8). The formation of formazan, which was reduced by the SOD activity of the cell-free extract, was monitored at 560 nm. Cell-free extracts were made using X-press (AB Biox, Göteborg, Sweden [29]) and their protein content was measured with Bradford’s reagent. Specific SOD activity was expressed as U/μg protein. Here, 1 U was defined as an amount of SOD that inhibited formazan formation by 50%.

### 2.3. Generation of Human Macrophages and Testing Susceptibility of Conidia by Macrophage Killing

Human heparinized leukocyte-enriched buffy coat samples were obtained from healthy blood donors. Peripheral blood mononuclear cells (PBMC) were isolated from buffy coats by Ficoll-Paque Plus (GE Healthcare, Little Chalfont, Buckinghamshire, UK) gradient centrifugation. PBMCs were used for primary monocyte separation by positive selection using CD14 microbeads (Miltenyi Biotech, Bergish Gladbach, Germany). For macrophage differentiation, freshly isolated monocytes were plated at a density of 10^6^ cells/mL in 24-well cell culture plates in RPMI 1640 medium (Sigma-Aldrich, St. Louis, MO, USA) supplemented with 10% heat-inactivated fetal bovine serum (FBS, Life Technologies Corporation, Carlsbad, CA, USA), 2 mM L-glutamine (Biosera, Nuaille, France), 100 U/mL penicillin (Biosera, Nuaille, France), and 100 μg/mL streptomycin (Biosera, Nuaille, France) for 5 days. The complete RPMI 1640 medium was supplemented with 80 ng/mL granulocyte–macrophage colony-stimulating factor (GM-CSF; Gentaur Molecular Products, London, UK) to generate an M1 macrophage phenotype and with 50 ng/mL macrophage colony-stimulating factor (M-CSF; PeproTech, Brussels, Belgium) to induce an M2 macrophage phenotype. On day 2 of the differentiation, half of the cell culture medium was replaced with fresh complete medium supplemented with the appropriate cytokines. During differentiation and stimulation, cells were incubated at 37 °C in a 5% CO_2_ humidified atmosphere. On day 5, cells were collected and, after determining the cell count, they were centrifuged at 400 G for 8 min. Following removal of the supernatant, fresh complete RPMI 1640 medium was added to the cells and then the macrophages were seeded at a density of 1 × 10^5^ cells/100 µL in 96-well cell culture plates. To each well, 100 µL of RPMI 1640 medium containing 1 × 10^5^ conidia was added. As controls, samples containing 100 µL of conidia suspension and 100 µL of RPMI 1640 medium (without macrophages) were also prepared. After incubation for 4 h at 37 °C in 5% CO_2_ humidified atmosphere, plates were centrifuged (400 G, 8 min), the supernatant was discarded, and macrophages were lysed by incubation with 200 µL of distilled water for 10 min. After serial dilutions, samples were assayed on Barratt’s minimal nitrate agar plates and colony forming units were determined after 36 h incubation at 37 °C. Both strains were tested with M-CSF and GM-CSF macrophages in 8-8 replicates.

### 2.4. Reverse-Transcriptional Quantitative Real-Time Polymerase Chain Reaction (RT-qPCR) Assays

Total RNA was isolated from lyophilized mycelial samples using Trisol reagent (Sigma-Aldrich, St. Louis, MO, USA) according to Chomczynski [31]. RT-qPCR assays were carried out using Xceed qPCR SG 1-step 2x Mix Lo-ROX kits (Applied Biotechnologies, Praha, Czech Republic) according to the manufacturer’s instructions. The list of the primer pairs is available in Table S1. The difference between the crossing points of the reference and target genes within a sample (ΔCP) was calculated and used as a measure of relative transcription. The AFUB_078400 gene (putative β-1,3-D-glucan synthase catalytic subunit) was selected as the reference gene.

### 2.5. High-Throughput RNA Sequencing

The following four types of culture were studied:

1. Cultures of the *A. fumigatus akuB^ku80^* reference strain;
2. Cultures of the *A. fumigatus* IP345 Δ*sodB* mutant;
3. Cultures of the *A. fumigatus akuB^ku80^* reference strain treated with 6 mM MSB at 20 h of cultivation;
4. Cultures of the *A. fumigatus* IP345 Δ*sodB* mutant treated with 6 mM MSB at 20 h of cultivation.

Mycelial samples (four culture types × three biological replicates) were taken at 20.5 h (half an hour after treatment). Total RNA was isolated as described above for RT-qPCR.

RNA sequencing was carried out at the Genomic Medicine and Bioinformatic Core Facility, Department of Biochemistry and Molecular Biology, Faculty of Medicine, University of Debrecen, Debrecen, Hungary. RNA libraries were made using a TruSeq RNA Sample preparation kit (Illumina, San Diego, CA, USA) following the manufacturer’s protocols. Library pools were sequenced (single-read 75 bp Illumina RNA sequencing) in the same lane of a sequencing flow cell yielding 16.6–29.2 million reads per sample.

In the case of each sample, more than 92% of the reads were aligned successfully to the genome of *A. fumigatus* A1163 (hisat2 version 2.1.0 [32]; https://fungidb.org/common/downloads/release-56/AfumigatusA1163/fasta/data/FungiDB-56_AfumigatusA1163_Genome.fasta (accessed on 11 March 2022); https://fungidb.org/common/downloads/release-56/AfumigatusA1163/gff/data/FungiDB-56_AfumigatusA1163.gff (accessed on 11 March 2022)). FeatureCounts (version 2.0.0) [33] was used to calculate count values, and DESeq2 (version 1.34.0) [34] was used to determine differentially expressed genes (DEGs; adjusted *p*-value < 0.05). RPKM (reads per kilobase per million mapped reads) values were calculated using the “rpkm” function of the edgeR package (version 3.36) [35]. Principle component analysis (PCA) was conducted with the “prcomp” function using the rlog(foldchange) values calculated by DESeq2.

### 2.6. Evaluation of Transcriptome Data

DEGs where log_2_FC > threshold, or log_2_FC < −1 threshold were regarded as upregulated and downregulated genes, respectively. Unless otherwise indicated, the threshold was 1. FC stands for “fold change”, and during the calculation of the log_2_FC values (DESeq2 software version 1.36.0), the untreated cultures (when the effects of the treatment was studied) or the cultures of the reference strain (when the consequences of the gene deletion was studied) were used as references.

The ShinyGO (http://bioinformatics.sdstate.edu/go/, accessed on 15 April 2024) platform was used for gene set enrichment analyses, applying the default settings. As the gene set enrichment analysis is highly dependent on the size of the gene sets examined, the calculations were performed with upregulated and downregulated gene sets determined using thresholds of 0, 0.5, and 1.

The enrichment of “Glycolysis”, “Antioxidant enzyme”, “Iron uptake”, “Siderophore cluster”, “Fe-S cluster assembly”, “Heme biosynthesis”, “Fe-S cluster protein”, “Heme binding protein”, and “Ribosome protein” genes in the upregulated and downregulated gene sets was also tested using the Fisher’s exact test (“fisher.test” function of R project; version 4.0.3). “Ribosome protein” genes of *A. fumigatus* Af293 were collected from the KEGG (Kyoto Encyclopedia of Genes and Genomes) pathway database (https://www.kegg.jp/pathway/afm03010, accessed on 15 April 2024). For the other gene groups the *A. fumigatus* Af293 gene lists described by Flipphi et al. [36] (“Glycolysis” genes), Emri et al. [37] (“Antioxidant enzyme”, “Iron uptake”, “Heme biosynthesis”, “Fe-S cluster assembly”, “Heme binding protein”, and “Fe-S cluster protein” genes), and Inglis et al. [38] (“Siderophore cluster” genes) were used. Orthologs of *A. fumigatus* Af293 in *A. fumigatus* A1163 were determined with OrthoMCL (version 2.0). In the pipeline, blast algorithms was used for the homology search, and the MCL (Markov cluster) algorithm [39,40] (www.micans.org/mcl/index.html, accessed on 30 May 2024) was used for clustering the orthologs that were found.

### 2.7. List of Abbreviations

DFP, deferiprone; ΔCP, difference between crossing points; DEG, differentially expressed gene; DCM, dry cell mass; Et, ethidium; Mn-SOD, manganese superoxide dismutase; MSB, menadione sodium bisulfite; PCA, principal component analysis; RNS, reactive nitrogen species; ROS, reactive oxygen species; RT-qPCR, reverse-transcriptional quantitative real-time polymerase chain reaction; RPKM, reads per kilobase per million mapped reads, wt, wild type

## 3. Results

**Figure 1:**
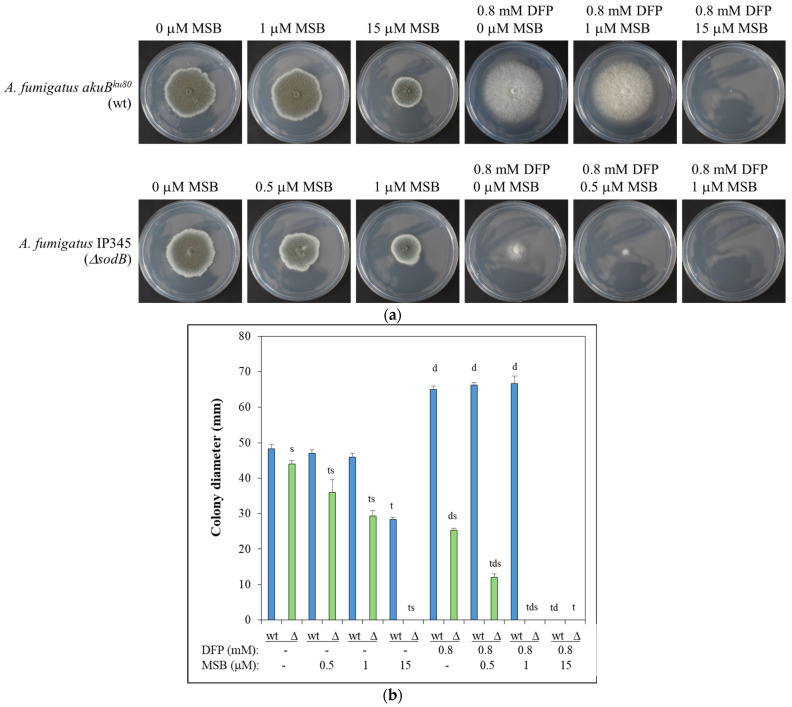
MSB stress sensitivity of *A. fumigatus* IP345 (Δ*sodB* gene deletion mutant; Δ; green) and *akuB^ku80^* (reference strain; wt; blue) on agar plates in the presence and absence of DFP iron chelator. (**a**) Five days old *A. fumigatus* cultures on agar plates. Representative photos are presented; the diameter of Petri dishes is 85 mm. (**b**) Diameter (mean ± SD, n = 3) of the colonies after 5 d of incubation. ^s,t,d^: Significant difference (Student’s *t*-test; *p* < 0.05) from the corresponding (s) reference strain, (t) MSB-untreated, and (d) DFP-untreated samples.

**Figure 2:**
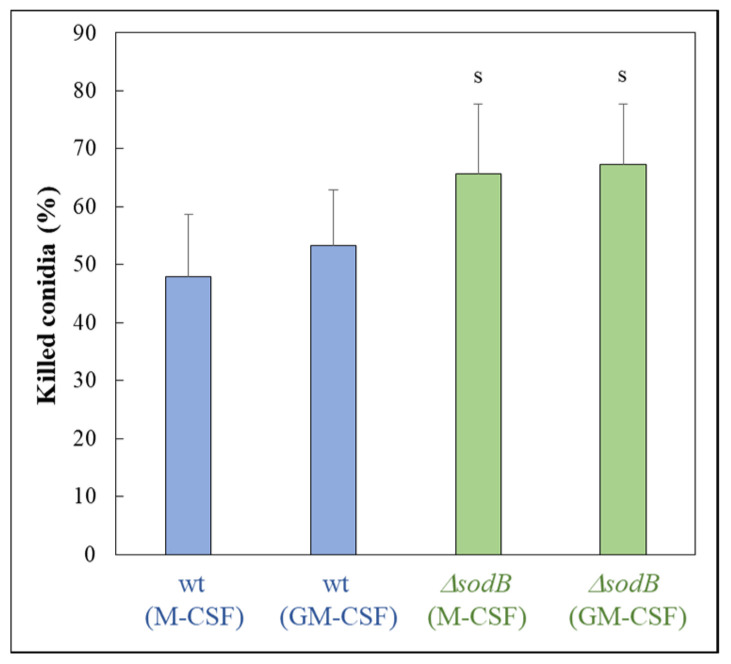
Susceptibility of *A. fumigatus* conidia (wt—*akuB^ku80^*, and Δ*sodB*—IP345) to killing by human macrophages (generated by M-CSF or GM-CSF). Mean ± SD (n = 8) are presented. ^s^—significant difference between the Δ*sodB* mutant and the reference strains with the same type of macrophages (Student’s *t*-test; *p* < 0.05).

The importance of mitochondrial Mn-SOD (SodB) during oxidative stress elicited by MSB treatment was shown in *A. fumigatus* using a *sodB* gene deletion mutant (IP345; Δ*sodB*) and the parental strain (akuB^ku80^; wild type; wt). The deletion of the *sodB* gene increased the MSB sensitivity (Figure 1) as expected. Treatment with the iron chelator DFP inhibited conidiogenesis of both strains (Figure 1a). Interestingly, this treatment increased the radial growth of the wt strain (Figure 1), suggesting that, similarly to carbon limitation [41], iron limitation also initiates this type of stress response in an attempt to escape from the nutrition-depleted environment. In contrast, deletion of the *sodB* gene reduced the DFP tolerance of the fungus (Figure 1). MSB sensitivity highly depended on the presence of DFP: treatment with DFP iron chelator increased MSB sensitivity in both strains (Figure 1). Deletion of the *sodB* gene slightly increased the susceptibility of conidia to killing by human M-CSF and GM-CSF macrophages (Figure 2).

**Figure 3:**
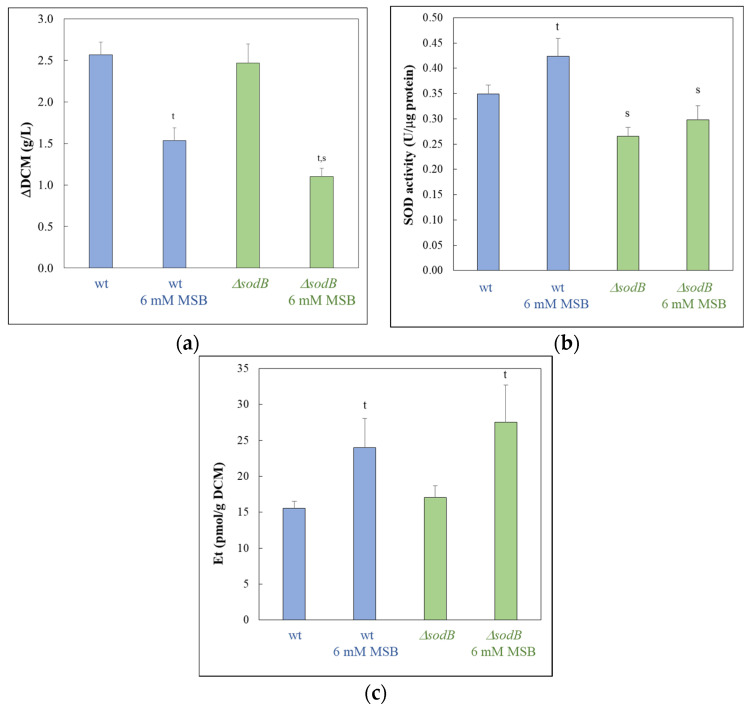
Changes in the DCM (**a**), specific SOD activities (**b**), and superoxide formation (**c**) after MSB treatment during submerged cultivation. *A. fumigatus* cultures (wt—*akuB^ku80^*, and Δ*sodB*—IP345) were treated with 6 mM MSB. Note, the composition of the medium used in these experiments was the same as that used for the surface cultures (except the agar content). Superoxide formation in *A. fumigatus* cultures was characterized with the formation of Et. Mean ± SD (n = 3) are presented. ^t^—significant difference between the MSB-treated and the appropriate untreated cultures (Student’s *t*-test; *p* < 0.05). ^s^—significant difference between the Δ*sodB* mutant and the *akuB^ku80^* reference strain under the same culturing conditions (Student’s *t*-test; *p* < 0.05). Note that the specific SOD activity of MSB-treated cultures of the Δ*sodB* strain did not differ significantly from that of untreated wt strain (Student’s *t*-test; *p* < 0.05).

Surprisingly, *A. fumigatus* tolerated MSB stress more in submerged cultures (Figure 3) (or on surface cultures, when MSB was added to 1 day-old cultures where conidia had already germinated; Figure S1) than on surface cultures (when conidia germinated in the presence of MSB) (Figure 1). In submerged cultures, MSB treatment increased superoxide formation and specific SOD activities in both strains (Figure 3b,c). The specific SOD activity of the Δ*sodB* mutant was lower than that of the wt strain irrespectively of the presence of MSB (Figure 3b), while the superoxide formation in the Δ*sodB* mutant exceeded that of the wt strain under MSB stress treatment (Figure 3c).

**Figure 4:**
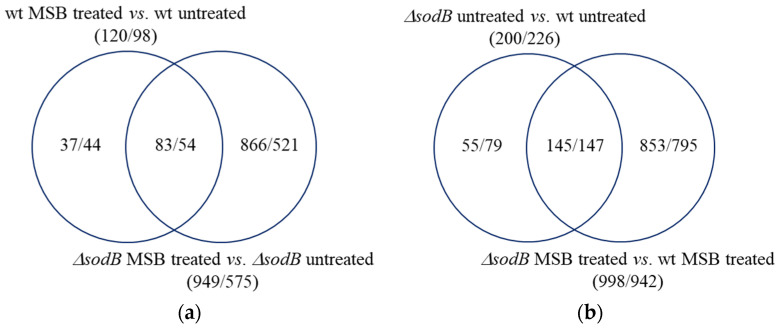
Venn analysis of the upregulated/downregulated genes. (**a**) Distribution of MSB stress responsive genes between the wt strain (*akuB^ku80^*) and the Δ*sodB* mutant (IP345). (**b**) Distribution of up-/downregulations caused by *sodB* gene deletion in untreated and 6 mM MSB-treated cultures. Upregulated and downregulated) genes were defined as DEGs where log_2_FC > 1 or log_2_FC < −1, respectively.

PCA implied that the three biological replicates had similar transcriptomes (Figure S2), and a good positive correlation was found between the RNA sequencing and the RT-qPCR data of the genes studied (Pearson’s correlation coefficient > 0.96; the number of studied genes was 14) (Table S1). The MSB treatment altered the transcriptome of both strains substantially (Figure 4a and Figure S2) and the transcriptomes of the wt and Δ*sodB* strains differed substantially under both untreated and MSB-treated conditions (Figure 4a and Figure S2).

The number of MSB stress-responsive genes was much higher in the Δ*sodB* gene deletion mutant than in the reference strain (Figure 4a). This concurs well with the elevated MSB stress sensitivity of the mutant found in both surface (Figure 1 [7]) and submerged cultures (Figure 3a). More than half of the MSB stress-responsive genes of the wt strain also responded to the MSB treatment in the Δ*sodB* mutant (Figure 4a). However, due to the large difference between the sizes of the two MSB stress responsive gene sets, many genes responded to MSB only in the mutant (Figure 4a). Moreover, the difference between the two strains under untreated conditions increased further after the treatment (Figure 4b). All these data imply that the two strains responded differently (i.e., with different genes) to the treatment, demonstrating that SodB was important for adaptation to the MSB-elicited oxidative stress.

**Figure 5:**
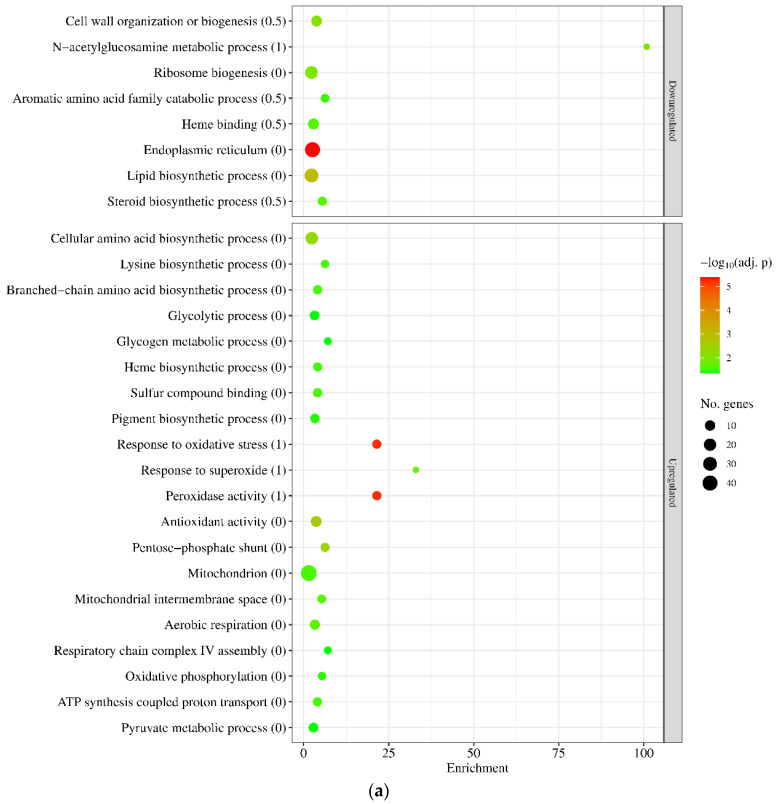

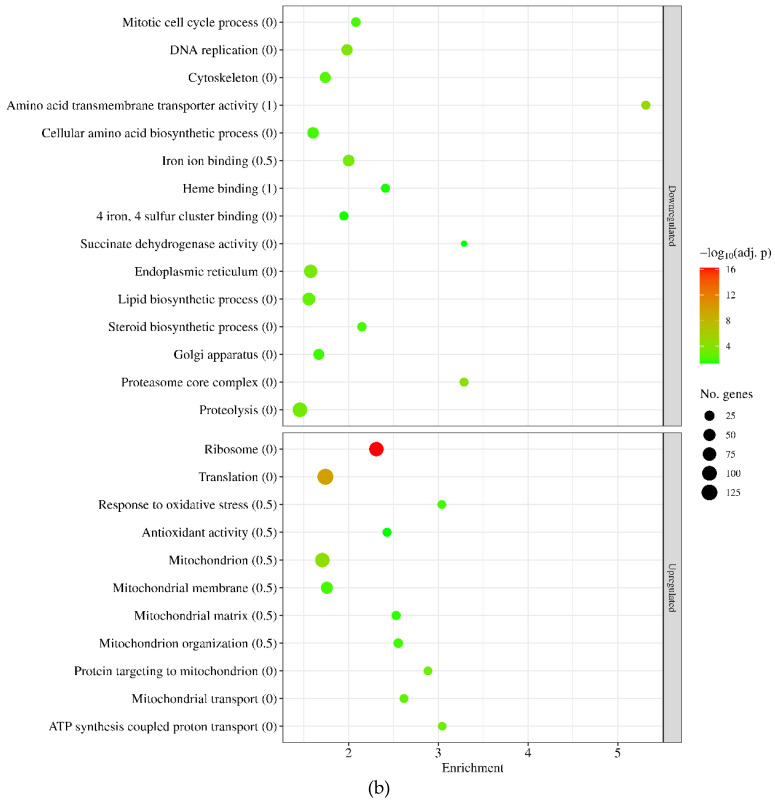
Genome-wide transcriptional consequences of MSB treatment studied by gene set enrichment analyses of upregulated and downregulated genes found with *A. fumigatus* wt (*akuB^ku80^*) (**a**) and Δ*sodB* (IP345) (**b**) strains. We present selected significantly enriched (*p* adjusted < 0.05) GO and KEGG pathway terms. The complete list of enriched terms is available in Table S2. Parenthesized numbers indicate gene set examined: “0”— all DEGs, “0.5”— DEGs with |log_2_FC| > 0.5, and “1”— DEGs with |log_2_FC| > 1. Only the set with the strongest criteria is displayed if a selected term was enriched in more than one gene set.

Interestingly, gene set enrichment analyses indicated that there were some similarities between the two stress responses (Figure 5 and Table S2). In both strains, genes of GO and KEGG pathway terms related to oxidative stress protection (like “Response to oxidative stress” and “Antioxidant activity”) or mitochondrial functions (like “Mitochondrion” and “ATP synthesis coupled proton transport”) were enriched in the upregulated gene set, and genes of “Heme binding”, “Endoplasmic reticulum”, “Lipid biosynthetic process”, and “Steroid biosynthesis” terms were enriched in the downregulated gene set (Figure 5 and Table S2). Genes related to vegetative growth (like “Cell wall organization and biogenesis” and “Ribosome biogenesis” in the wt strain and “Mitotic cell cycle” and “DNA replication” in the mutant) were also enriched in the downregulated gene set (Figure 5, Table S2), well in line with the growth slowdown detected after MSB treatment (Figure 3a). Regarding the “Heme binding” term, out of the 108 genes belonging to this gene group, 13 showed downregulation (DEGs with log_2_FC < −0.5) in the wt strain, while 45 did in the Δ*sodB* mutant (Table S2). In the case of “Endoplasmic reticulum” genes, out of the 150 genes, 40 and 72 genes were downregulated (DEGs), while in the case of “Mitochondrion” genes, out of the 297 genes, 48 and 139 genes were upregulated (DEGs) in the wt and the mutant strain, respectively (Table S2). These figures suggest that although similar functions were regulated in both strains by MSB treatment, the intensity of the response was larger in the Δ*sodB* mutant. The differences between the downregulated growth-related terms (cell wall and ribosome biogenesis in the wt strain vs. mitotic cell cycle and replication in the mutant) also implies differences in the intensity rather than nature of the responses.

Among the functional differences between the stress responses of the two strains, revealed by gene set enrichment analyses, the translation-related genes are noteworthy: “Ribosome biogenesis” genes were significantly enriched among the downregulated genes of the wt strain, while “Translation” genes were significantly enriched among the upregulated genes of the Δ*sodB* mutant (Figure 5 and Table S2). Enrichment of the “Mitochondrion”, “Ribosome biogenesis”, and “Translation” genes within the upregulated gene set was observed in both untreated and MSB-treated cultures when the transcriptomes of the two strains were directly compared (Table S2). Interestingly, “Heme binding” genes were enriched inside the upregulated gene set when comparing the transcriptome of untreated cultures (Δ*sodB* untreated vs. wt untreated), while these genes together with the “4 iron, 4 sulfur cluster binding” genes were enriched inside the downregulated gene set when examining MSB-treated cultures (Δ*sodB* MSB-treated vs. wt MSB-treated) (Figure S3 and Table S2).

**Figure 6:**
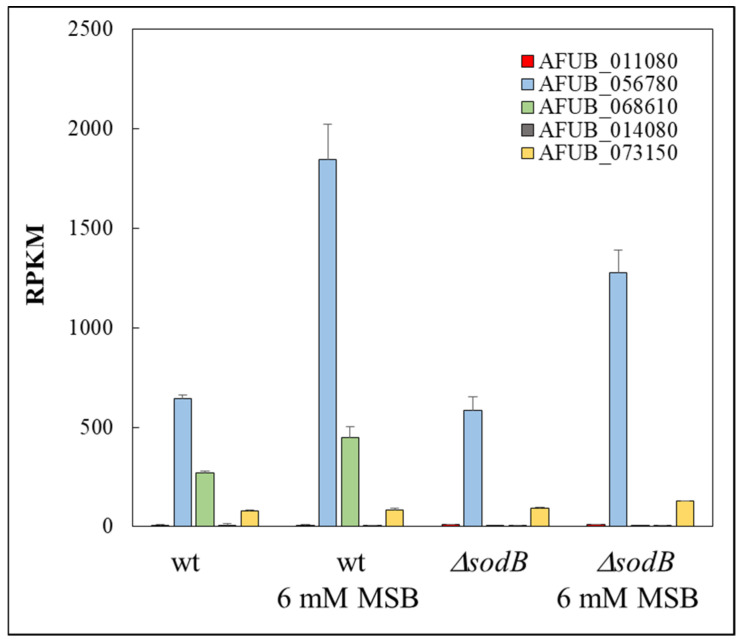
Transcription (RPKM) values of known and putative SOD genes in the *A. fumigatus* wt (*akuB^ku80^*) and Δ*sodB* (IP345) strains. AFUB_056780, AFUB_068610 (*sodB*), AFUB_014080, and AFUB_073150 are the orthologs of *A. fumigatus* Af293 *sod1*, *sod2*, *sod3*, and *sod4* genes, respectively.

The transcriptional behavior of a subset of genes (“Glycolysis genes”, “Antioxidant enzyme genes”, “Iron uptake genes”, “Siderophore cluster genes”, “Fe-S cluster assembly genes”, “Heme biosynthesis genes”, “Fe-S cluster protein genes”, “Heme binding protein genes”, and “Ribosome protein genes”) was also evaluated in detail (Table S3). “Antioxidant enzyme genes” showed enrichment in the upregulated gene sets of both strains after MSB exposure, as expected (Table S3). “Glycolysis genes” were also among the genes enriched in the upregulated gene set by MSB treatment in both strains, suggesting increased ATP demand in the stressed cultures (Table S3). Many more genes were significantly upregulated (22 vs. 12) in the Δ*sodB* mutant than in the wt strain (Table S3). The *sodB* gene was upregulated by MSB exposure in the wt strain suggesting its importance to protect mitochondria under MSB stress (Figure 6 and Table S3). However, the missing SodB was not compensated by increased transcription of any SOD genes (Figure 6 and Table S3). Only the transcription of AFUB_073150 gene (an ortholog of sod4 essential SOD gene of *A. fumigatus* Af293 [7] showed a moderate increase (Figure 6 and Table S3).

The iron metabolism of the two strains responded to MSB stress in a different manner (Table S3). The upregulated gene set of the Δ*sodB* mutant was enriched for iron acquisition genes, including genes involved in reductive iron assimilation (RIA) and siderophore-mediated pathways (Table S3). These changes were accompanied by the upregulation of the hapX (positive regulator of iron uptake [42]) ortholog AFUB_052420 and the downregulation of the sreA (negative regulator of iron uptake [42]) ortholog AFUB_058830 (Table S3). “Fe-S cluster assembly genes” were also upregulated in the mutant (Table S3). Despite the fact that that the “Fe-S cluster protein genes” of the Δ*sodB* mutant were enriched neither in the upregulated nor in the downregulated gene set (Table S3), the majority of “Fe-S cluster protein genes” altered their transcription significantly in response to MSB treatment (Table S3). Similar bulk changes in these genes were not observed in the wt strain (Table S3). In contrast, only the upregulated gene set of the wt strain was enriched for “Heme biosynthesis genes” under MSB stress (Table S3), while the downregulated gene set of both strains was enriched for “Heme binding protein genes” and, as mentioned above, the number of downregulated “Heme binding protein genes” was much greater in the case of the Δ*sodB* mutant than in the wt strain (Table S3). Both mitochondrial and cytosolic “Ribosome protein genes” showed bulk upregulation in the Δ*sodB* mutant relative to the wt strain even under untreated conditions, and MSB treatment further increased their transcription (Table S3). These alterations may indicate that resynthetizing oxidative stress damaged proteins (e.g., Fe-S cluster proteins) is crucial for the Δ*sodB* mutant to adapt MSB stress.

## 4. Discussion

A large difference between the MSB susceptibilities of surface and submerged *A. fumigatus* cultures were found (Figure 1 and Figure 2), and this difference was even larger in the case of the Δ*sodB* mutant than with the wt strain (Figure 1 and Figure 2). Similar phenomenon was observed previously with *A. nidulans* wild type and Δ*sodB* gene deletion mutant strains [11]. This may be the consequence of the reduced oxygen availability of submerged cultures, which reduced the superoxide generation of MSB, or, alternatively, this feature of the strains may be explained by the outstanding importance of mitochondria during the germination of conidia. The results of well-diffusion assays (Figure S1) supported this assumption: the wt strain showed reduced susceptibility to MSB when conidia germinated in the presence of MSB in comparison to those experiments when MSB was added to one-day-old cultures after conidia had already germinated (Figure 1 and Figure S1). *A. fumigatus* enters the human body by inhalation as conidia and most of them are eliminated by phagocytes via both oxidative and non-oxidative defense mechanisms [43,44,45]. The high sensitivity of (germinating) conidia to oxidative stress compared to hyphae support the view that successful germination is crucial for efficient infection, and antifungal strategies based on the disruption of germination can be beneficial [46]. The importance of these observations was also underlined in previous experiments demonstrating that conidia successfully germinated under oxidative stress had an increased ability to adapt to iron limitation conditions [47], which often occur in the human body. The stress tolerance attributes of conidia, however, also depend on the stresses that the conidia-forming colony is exposed to [48,49,50,51]. Any conditions that increase the oxidative stress tolerance of conidia produced in our environment may also increase the risk of successful infections, which should be taken into consideration when we are aiming to design healthy (indoor) environments for ourselves.

Previously, we found that MSB-induced stress responses of the *A. nidulans* wt and Δ*sodB* gene deletion strains were very similar to each other in both type and intensity [11]. These responses were primarily based on a reduction in the intensity of mitochondrial function, removal of damaged mitochondria, and degradation and resynthesis of damaged mitochondrial proteins [11]. This suggests that SodB, even though the *sodB* gene was upregulated in the wt strain, was unable to effectively protect mitochondria under the conditions studied. In contrast, deletion of *sodB* in *A. fumigatus* largely altered the MSB induced oxidative stress response and was based mainly on the upregulation of mitochondrial functions (Figure 4, Figure 5 and Figure S1). Upregulation of *sodB* by MSB exposure in the wt *A. fumigatus* strain (Figure 6, Table S3) and the increased growth reduction observed in the Δ*sodB* mutant in comparison to the wt strain (Figure 3a) also emphasize the importance of SodB in oxidative stress protection. Nevertheless, the lack of SodB did not initiate alternative stress protection mechanisms in the mutant strain; instead, the difference between the oxidative stress responses of the wt strain and the Δ*sodB* mutant manifested mainly in the intensity of the response. As a consequence, although we found more treatment-responsive genes in the Δ*sodB* mutant than in the wt strain (Figure 4) most of these genes belonged to the same or similar biological processes as the treatment-responsive genes in the wt strain (Figure 5 and Figure S2, Tables S2 and S3). These data suggest that SodB was able to considerably reduce the adverse effects of MSB on mitochondria in this case. The molecular background of the difference between *A. nidulans* and *A. fumigatus* is not known and may not be directly related to SodB or its regulation. For example, an efficient detoxification of MSB by *A. fumigatus* mycelia would reduce the effect of this stressor to a level that SodB is able to handle. However, it was unexpected that 6 mM MSB was required to inhibit the growth of *A. fumigatus* mycelium (Figure 3a) to a level comparable to the effect of 0.16 mM MSB on *A. nidulans* mycelium [11]. This shows a significant difference between the two species, which may also contribute to their different virulence.

Nevertheless, the substantial transcriptional changes in iron metabolism accompanied with upregulation of “Ribosome protein” genes (Figure 5, Tables S2 and S3) observed only in the Δ*sodB* gene deletion mutant are notable. These transcriptional changes suggest that the elevated superoxide level (Figure 3c) generated by MSB treatment may have destroyed Fe-S cluster proteins of mitochondria in the absence of SodB-mediated protection. Previous studies demonstrated that MSB generates superoxide radical anions in mitochondria as well [52], and Fe-S clusters are rather sensitive to this type of ROS [53]. Re-synthesis of Fe-S cluster (and other damaged mitochondrial) proteins requires enhanced translation, as indicated by upregulated ribosome protein genes in the Δ*sodB* mutant (Figure 5, Tables S2 and S3), and the increased iron demand of Fe-S cluster reconstitution and/or de novo synthesis changed the iron metabolism considerably (Table S3). For example, upregulations of both the RIA pathway and siderophore-mediated iron uptake genes, as well as Fe-S cluster assembly genes, were observed in the gene deletion mutant (Table S3). In order to conserve iron, the cells reduced the production of several heme-binding and unnecessary Fe-S cluster proteins (Table S3). We hypothesize that efficient and economical iron metabolism in *A. fumigatus* is essential for the fungus to survive mitochondria-destroying stresses. The increased DFP sensitivity of the Δ*sodB* mutant may also be a consequence of the increased turnover of iron-dependent proteins due to the lack of SodB (Figure 1).

Importantly, *A. fumigatus* must cope with oxidative stress under iron-limited conditions in the human body [54]. Previous experimental data demonstrated that iron limitation increases the oxidative stress sensitivity of the fungus [27,55]. Triple deletion of *sod1, sod2,* and *sod3* genes did not decrease the virulence of *A. fumigatus* in an immunocompromised murine aspergillosis model; however this triple mutant exhibited increased sensitivity to killing by alveolar macrophages of immunocompetent mice [7]. In our experiments, the Δ*sodB* mutant showed a moderately increased susceptibility to human GM-CSF and M-CSF macrophages (Figure 2), supporting the view that not only cytosolic SodA (CuZn-SOD) but also SodB contributes to survival in phagolysosomes [56]. As our results suggest, the minor effect of the lack of SodB on survival in macrophages might be explained, at least partially, with the highly efficient and economical iron metabolism of the fungus. In the opportunistic human pathogenic yeast *Candida albicans*, the orthologous Sod2p did not contribute to the in vivo virulence [57]. Meanwhile, the Δ*sodB* mutant of *Cryptococcus neoformans* was avirulent [58]. The unchanged virulence of the Δ*sod1*Δ*sod2*Δ*sod3 A. fumigatus* strain [7] and the only moderate increase found in the susceptibility of the Δ*sodB* mutant to killing by macrophages (Figure 2) suggest that any antifungal therapy solely aiming at the inactivation of Mn-SOD in *A. fumigatus* would be inefficient. However, such treatment may enhance the efficacy of antifungal therapies based on the inhibition of iron metabolism [59], as inhibition of Mn-SOD, in addition to inducing oxidative stress, indirectly increases iron demand through the need to resynthesize damaged Fe-S cluster proteins.

## Data Availability

The transcriptome data sets are available in the Gene Expression Omnibus database (GEO; http://www.ncbi.nlm.nih.gov/geo/, accessed on 1 September 2024) with the following accession number: GSE269970.

## Supplementary Materials

The following supporting information can be downloaded at: https://www.mdpi.com/article/10.3390/jof10120823/s1, Figure S1: Effect of MSB treatment on *A. fumigatus* (*akuB^ku80^*; wt reference strain and IP345 Δ*sodB* gene deletion mutant) mycelium and conidia in surface cultures; Figure S2: Principal component (PC) analysis of the RNAseq data (rlog values generated by the DESeq2 software); Figure S3: Genome-wide transcriptional consequences of *sodB* gene deletion studied by gene set enrichment analyses of upregulated and downregulated genes found with untreated (A) and MSB-treated (B) cultures; Table S1: RT-qPCR data of selected genes; Table S2: Results of gene set enrichment analyses; Table S3: Transcriptional behavior of selected gene groups.
