# Supplementary material for: Resynthesis of Damaged Fe-S Cluster Proteins Protects Aspergillus fumigatus Against Oxidative Stress in the Absence of Mn-Superoxide Dismutase
Source: J Fungi (Basel). 2024 Nov 27;10(12):823. doi: 10.3390/jof10120823 (PMC11677433; doi:10.3390/jof10120823)
Supplement: Supplementary file 1 [file jof-10-00823-s001.zip › Figure S1.pptx]

## Slide 1
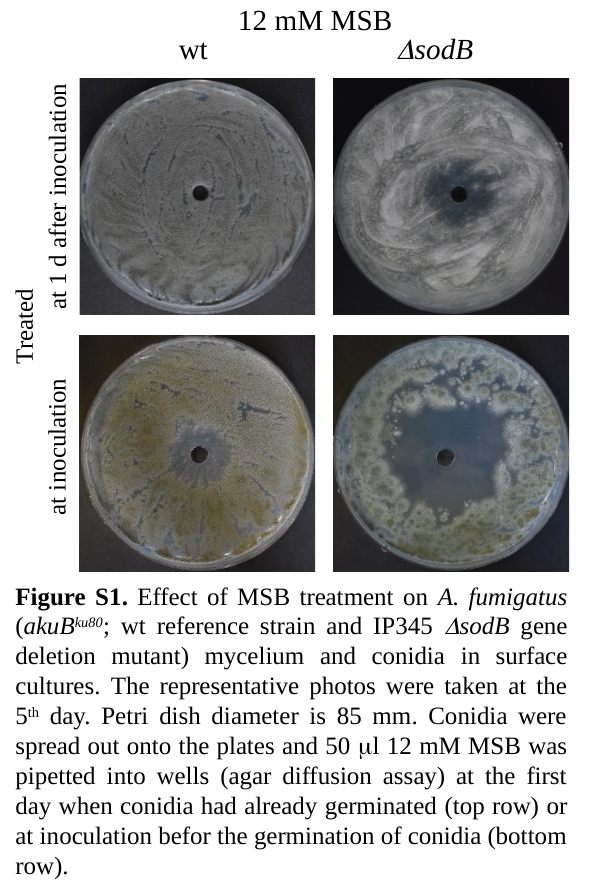

12 mM MSB
wt DsodB
at 1 d after inoculation
Treated
at inoculation
Figure S1. Effect of MSB treatment on A. fumigatus (akuBku80; wt reference strain and IP345 DsodB gene deletion mutant) mycelium and conidia in surface cultures. The representative photos were taken at the 5th day. Petri dish diameter is 85 mm. Conidia were spread out onto the plates and 50 ml 12 mM MSB was pipetted into wells (agar diffusion assay) at the first day when conidia had already germinated (top row) or at inoculation befor the germination of conidia (bottom row).
