# Supplementary material for: Resynthesis of Damaged Fe-S Cluster Proteins Protects Aspergillus fumigatus Against Oxidative Stress in the Absence of Mn-Superoxide Dismutase
Source: J Fungi (Basel). 2024 Nov 27;10(12):823. doi: 10.3390/jof10120823 (PMC11677433; doi:10.3390/jof10120823)
Supplement: Supplementary file 1 [file jof-10-00823-s001.zip › Figure S2.pptx]

## Slide 1
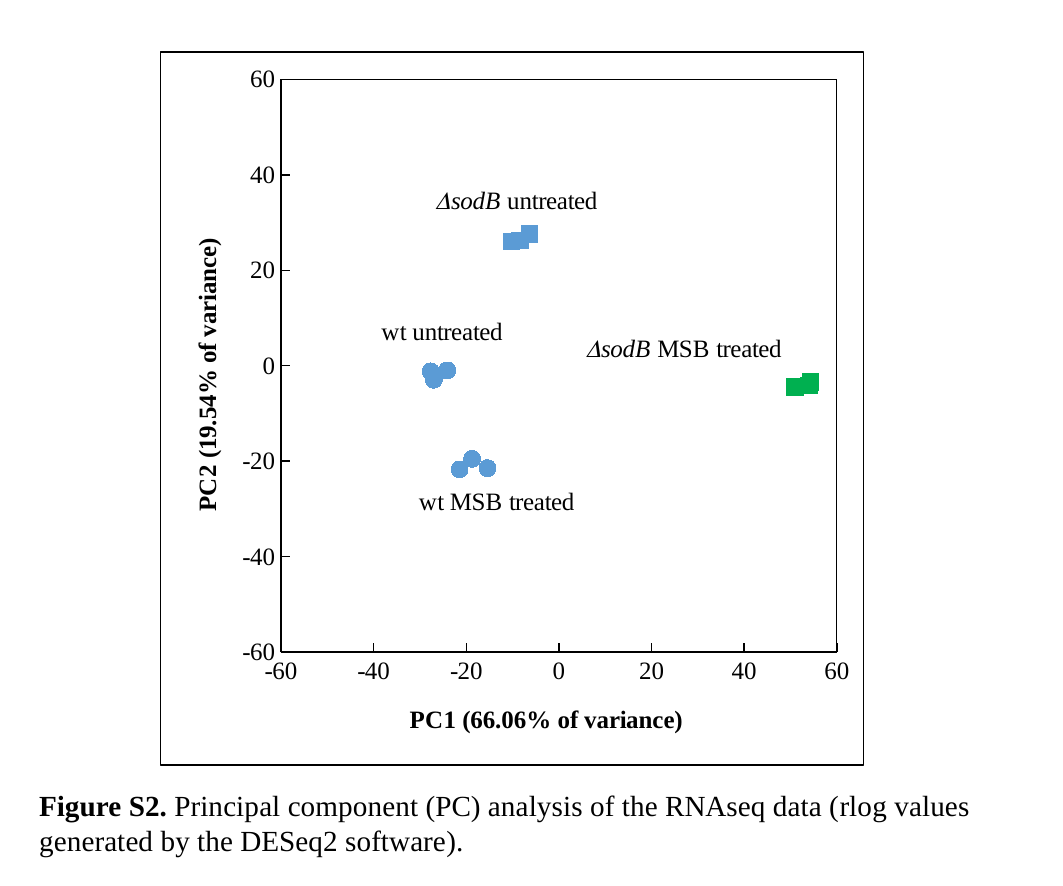

### Chart
| Category | PC2 |
|---|---|Figure S2. Principal component (PC) analysis of the RNAseq data (rlog values generated by the DESeq2 software).
