# Supplementary material for: Resynthesis of Damaged Fe-S Cluster Proteins Protects Aspergillus fumigatus Against Oxidative Stress in the Absence of Mn-Superoxide Dismutase
Source: J Fungi (Basel). 2024 Nov 27;10(12):823. doi: 10.3390/jof10120823 (PMC11677433; doi:10.3390/jof10120823)
Supplement: Supplementary file 1 [file jof-10-00823-s001.zip › Figure S3.pptx]

## Slide 1
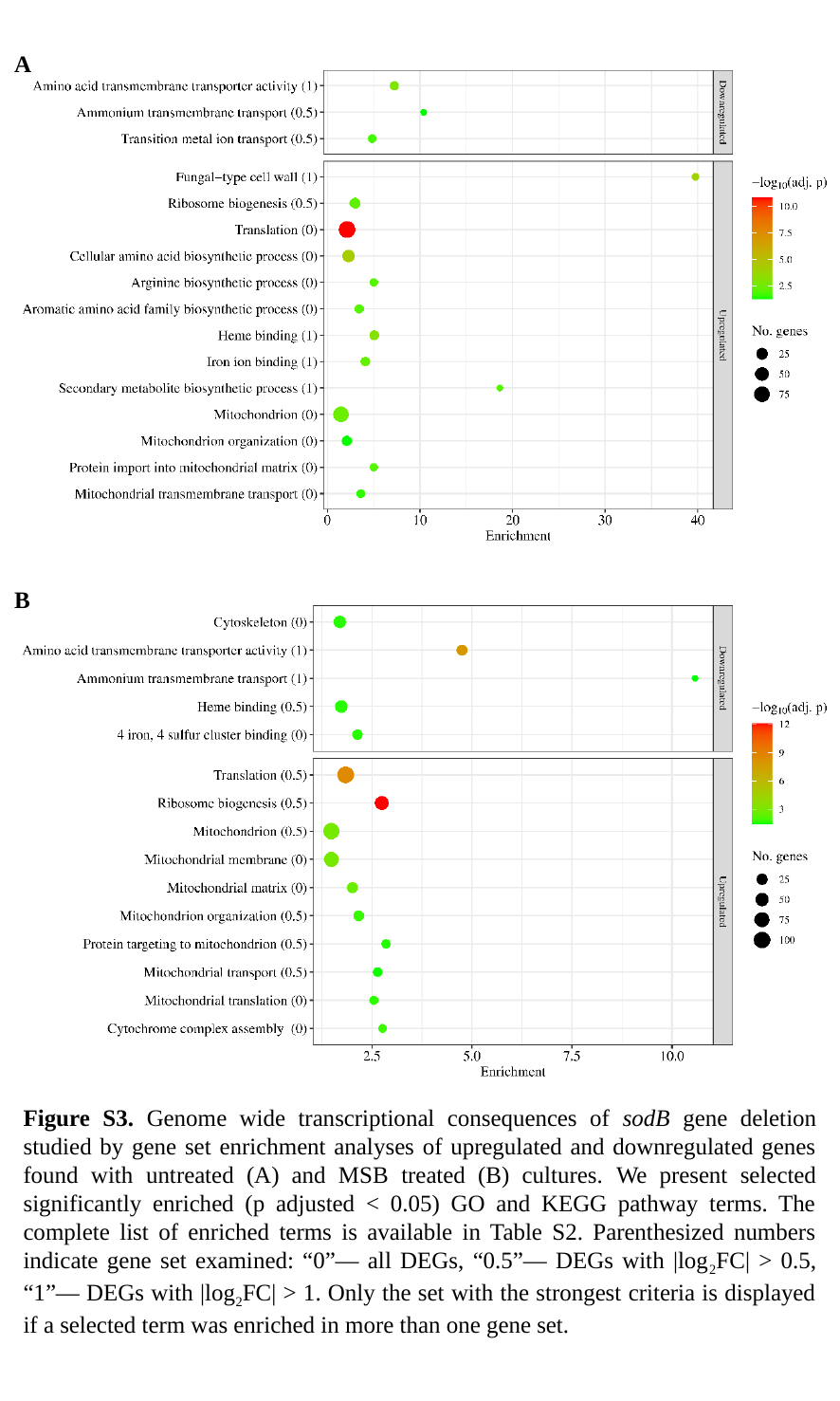

A
B
Figure S3. Genome wide transcriptional consequences of sodB gene deletion studied by gene set enrichment analyses of upregulated and downregulated genes found with untreated (A) and MSB treated (B) cultures. We present selected significantly enriched (p adjusted < 0.05) GO and KEGG pathway terms. The complete list of enriched terms is available in Table S2. Parenthesized numbers indicate gene set examined: “0”— all DEGs, “0.5”— DEGs with |log2FC| > 0.5, “1”— DEGs with |log2FC| > 1. Only the set with the strongest criteria is displayed if a selected term was enriched in more than one gene set.
